# Trial-based Cost-effectiveness Analysis of an Immediate Postoperative Mitomycin C Instillation in Patients with Non–muscle-invasive Bladder Cancer

**DOI:** 10.1016/j.euros.2021.12.008

**Published:** 2022-01-17

**Authors:** Anouk E. Hentschel, Christian J. Blankvoort, Judith Bosschieter, André N. Vis, R. Jeroen A. van Moorselaar, Judith E. Bosmans, Jakko A. Nieuwenhuijzen

**Affiliations:** a Department of Urology, Amsterdam University Medical Centers, Vrije Universiteit Amsterdam, Cancer Center Amsterdam, Amsterdam, The Netherlands; b Department of Health Sciences, Faculty of Science, Vrije Universiteit Amsterdam, Amsterdam Public Health Research Institute, Amsterdam, The Netherlands

**Keywords:** Cost-effectiveness, Mitomycin C, Non–muscle-invasive bladder cancer, Intravesical chemotherapy

## Abstract

**Background:**

Bladder cancer imposes a significant public health burden on the European Union. There is a need for cost-effective treatment and follow-up regimens.

**Objective:**

To assess the cost-effectiveness of immediate mitomycin C (MMC) instillation within 1 d after surgery compared to delayed MMC instillation within 2 wk after surgery with further adjuvant treatment, depending on the patient’s risk group.

**Design, setting, and participants:**

This economic evaluation was based on a randomized controlled trial among 2243 Dutch patients with non-muscle-invasive bladder cancer (NMIBC) patients from a health care perspective over a 3-yr time period.

**Outcome measurements and statistical analysis:**

The treatment effect was measured as time to recurrence and recurrence-free survival. Missing effect data were imputed with multiple imputation. Health care utilization and related costs were estimated on the basis of treatment protocols for NMIBC patients in the Netherlands. Statistical uncertainty was estimated using bootstrapping and is graphically presented using cost-effectiveness planes and cost-effectiveness acceptability curves.

**Results and limitations:**

Time to recurrence was significantly longer for immediate MMC instillation (27.31 mo) than for delayed MMC instillation (24.97 mo), with an adjusted mean difference of 2.21 mo (95% confidence interval [CI] 1.58–2.84). The proportion of patients with recurrence-free survival was significantly higher after immediate MMC instillation (0.65) than after delayed MMC instillation (0.56), with an adjusted mean difference of 0.08 (95% CI 0.06–0.11). Total mean health care costs per patient were significantly lower for immediate MMC instillation (€22 959) than for delayed MMC instillation (€24 624), with an adjusted mean difference of −€1350 (95% CI −€1799 to −€900). The study is limited by the retrospective estimation of costs.

**Conclusions:**

This trial-based cost-effectiveness analysis shows that from a health care perspective, immediate MMC instillation is more effective and less expensive compared to delayed MMC instillation.

**Patient summary:**

We assessed the cost-effectiveness of immediate bladder instillation of mitomycin C after surgery to reduce the risk of recurrence after removal of the bladder tumor as compared to delayed instillation in a large Dutch population of patients with non–muscle-invasive bladder cancer. We found that immediate instillation was more effective and less expensive than delayed instillation. We conclude that immediate mitomycin C instillation is a cost-effective treatment for non–muscle-invasive bladder cancer.

## Take Home Messages

This trial-based cost-effectiveness analysis demonstrates that from a health care perspective, immediate mitomycin C instillation is a cost-effective treatment regimen for non-muscle-invasive bladder cancer (NMIBC) compared to delayed mitomycin C instillation.

## 1 Introduction

In 2012, health care costs related to bladder cancer (BC) in the EU were estimated at €2.9 billion and accounted for 5% of total cancer-related health care costs [1]. Since BC-related health care costs are expected to rise further in the future, it is important to implement cost-effective treatment and follow-up regimens [2], [3].

Non–muscle-invasive bladder cancer (NMIBC) is classified as low, intermediate, or high risk according to the European Association of Urology (EAU) guidelines [4]. After transurethral resection of bladder tumor (TURBT), patients receive a single chemotherapeutic instillation if the tumor is papillary NMIBC (and bladder perforation/extensive bleeding are absent) [4]. Patients classified as having intermediate or high risk are treated afterwards with an adjuvant treatment schedule. Despite adjuvant treatment, NMIBC commonly recurs or progresses, and hence intensive follow-up and reinterventions involve high health care costs after initial treatment [3].

For NMIBC patients, an immediate postoperative chemotherapeutic instillation might be a cost-effective treatment compared to deferred instillation [3], [5]. Our previous randomized controlled trial (RCT) in 2243 patients demonstrated that an immediate postoperative mitomycin C (MMC) instillation effectively reduced the risk of recurrence (*p* < 0.001) in comparison to delayed postoperative MMC instillation. The beneficial effect on recurrence was observed regardless of administration of further adjuvant instillations [5]. However, the risk groups in that trial did not match current EAU guidelines [4], [5]. Therefore, patients were reclassified into EAU risk groups to evaluate whether immediate postoperative MMC instillation was beneficial for NMIBC patients across all EAU risk groups. This analysis showed that all NMIBC subgroups benefit from immediate postoperative MMC instillation [6].

Several model-based analyses have shown that immediate postoperative MMC instillation results in cost savings [7], [8], [9], [10]. To the best of our knowledge, the cost-effectiveness of immediate postoperative MMC instillation has not yet been assessed in a trial-based analysis. Therefore, we conducted an economic evaluation based on a RCT comparing immediate postoperative MMC instillation to delayed MMC instillation, with further adjuvant treatment depending on the patient’s risk group.

## 2 Patients and methods

In the RCT, 2243 NMIBC patients from 63 Dutch hospitals were included between 1998 and 2003 [5]. The Medical Ethical Committee of Amsterdam UMC (location VUmc) approved the trial and written informed consent was obtained from all patients. All patients were included in this post hoc economic evaluation, which was conducted from a health care perspective over a 3-yr time period.

### 2.1 Patients and randomization

Patients were recruited by their local physicians if their cystoscopy was suggestive of NMIBC, if they were aged ≥18 yr, and if their health status allowed for 3-yr follow-up. Patients had to be diagnosed with non–muscle-invasive urothelial carcinoma of the bladder without evidence of a tumor location elsewhere in the urinary tract or a concurrent malignancy outside the urinary tract. The bladder tumor had to be completely resected. The exclusion criteria were systemic chemotherapy or immunotherapy, previous radiotherapy, or chemotherapeutic instillations within 3 yr before inclusion, as well as laboratory abnormalities (leukopenia, thrombocytopenia, and abnormal urea/creatinine) and severe urinary tract infections. Before surgery, patients were allocated to immediate MMC instillation within 1 d after surgery or to delayed MMC instillation within 2 wk after surgery. Patients received 40 mg of MMC in 50 ml of saline (0.9%). After surgery, patients were classified as having low, intermediate, or high risk according to their tumor characteristics.

### 2.2 Treatment and follow-up

**Fig. 1:**
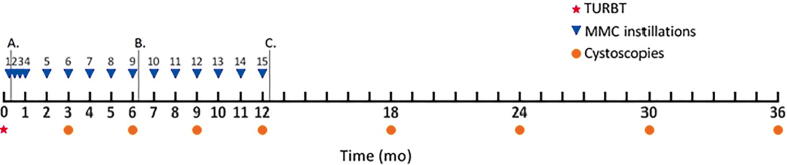
Timing of mitomycin C (MMC) instillations and cystoscopies during the 3-yr (36 mo) follow-up period. Patients with non–muscle-invasive bladder cancer in the (A) low, (B) intermediate, and (C) high risk group received a total of one, nine, and 15 MMC instillations, respectively. The timing and number of follow-up cystoscopies were similar for all three risk groups. TURBT = transurethral resection of bladder tumor.

Patients in the low-, intermediate-, and high-risk groups received a total of one, nine, and 15 MMC instillations, respectively (Fig. 1). In accordance with the trial protocol, NMIBC patients received no other types of instillation besides MMC. Bacillus Calmette-Guérin (BCG) instillations, which are now commonly administered to patients with high-risk NMIBC, were therefore not administered [4], [5]. The timing and number of follow-up cystoscopies were similar for all three risk groups: every 3 mo in the first year, and every 6 mo in the second and third years (Fig. 1). After initial TURBT, histological evidence of BC was considered as recurrence, and histological evidence of muscle-invasive BC (MIBC) as progression. Follow-up was terminated after a first recurrence. Patients were considered censored if they were lost to follow-up without experiencing recurrence or if they died. Small recurrences were treated with electrocauterization and others with TURBT [5].

Since costs were estimated retrospectively in this post hoc analysis, treatment and follow-up were assumed to be in accordance with the trial protocol. For patients who experienced a recurrence, we made the following assumptions about the course of events after the recurrence, as a recurrence itself was the endpoint of the study: treatment and follow-up after recurrence were in accordance with the EAU guidelines, patients showed up at all treatment and follow-up visits, patients did not experience a second recurrence, and patients survived the 3-yr follow-up period [4]. However, these assumptions may differ from reality, which could influence both the costs and the effects of the therapies investigated. Disease could recur as intermediate- or high-risk NMIBC or could progress to MIBC. Supplementary Table 1 summarizes the assumptions regarding the treatment and follow-up schedules among NMIBC patients with a recurrence [4].

### 2.3 Economic evaluation

#### 2.3.1 Effect measures

Treatment effects included time to recurrence and recurrence-free survival. Time to recurrence was the time between initial TURBT and first histological evidence of recurrence. Recurrence-free survival was the proportion of patients without recurrence over the 3-yr follow-up period.

#### 2.3.2 Cost measures

Costs were estimated from a health care perspective from initial TURBT until 3 yr later [5]. Since costs were not prospectively collected at a patient level during the trial, costs had to be estimated based on the treatment and follow-up schedules described in Figure 1 and Supplementary Table 1. None of the patients required surgical intervention or hospital admission for adverse events during the trial. Therefore, associated costs were considered negligible and were thus excluded [5].

To value health care utilization, Dutch standard costs were used if available [11]. Otherwise, we used mean prices for diagnosis-treatment combinations from the Dutch Health Care Authority [12]. Consumer price indices were used to adjust costs for inflation [13]. Costs per unit of health care utilization are summarized in Table 1.

**Table 1:** Costs per unit of health care utilization used in this economic evaluation

Health care utilization	Cost per unit (€)
Transurethral resection of bladder tumor	3772 [11]
Hospitalization after TURBT	495 [12]
Immediate mitomycin C instillation	838 [11]
Delayed mitomycin C instillation	838 [11]
Cystoscopy	645 [11]
Adjuvant instillation	838 [11]
Electrocauterization	1218 [11]
Cystectomy with bilateral lymph node dissection	21 729 [11]
Hospitalization after cystectomy with bilateral lymph node dissection	6140 [11]
Computed tomography scan	645 [11]

### 2.4 Statistical analyses

Analyses were conducted according to the intention-to-treat principle [14]. Baseline characteristics are described using the median and interquartile range (IQR) for continuous data, and the frequency and percentage for categorical data. The Mann-Whitney U test was used to compare means for continuous data between the treatment arms. The χ^2^ test was used to compare categorical data between the treatment arms.

#### 2.4.1 Missing data

Effect data were imputed for patients who did not complete the 3-yr follow-up period and who did not experience an event of interest before dropping out. Missing effect data were imputed by treatment arm with multiple imputation via chained equations. The imputation model contained all variables included in the analysis models, and variables that differed between patients with and without missing data [15]. Predictive mean matching was used to deal with skewed cost data [16]. A total of 15 data sets were needed for a loss of efficiency of <5%. Rubin’s rules were used to pool the results for the imputed data sets [15].

#### 2.4.2 Cost-effectiveness analyses

In the second and third follow-up years, costs were discounted at an annual rate of 4.0% and effects at an annual rate of 1.5% [11], [17]. Differences in costs and effects between the treatment arms were estimated using bivariate linear regression analyses [18]. Analyses were adjusted for possible confounders: age, concomitant carcinoma in situ, gender, primary/recurrence, tumor grade, tumor multiplicity, and tumor stage [4]. Bias-corrected and accelerated bootstrapping with 5000 replications was performed to estimate 95% confidence intervals (CIs) for the cost and effect differences.

Incremental cost-effectiveness ratios (ICERs) were calculated by dividing the difference in costs between immediate and delayed MMC instillation by the difference in effects. For time to recurrence, the ICER indicates the costs associated with one additional recurrence-free month for immediate versus delayed MMC instillation. For recurrence-free survival, the ICER indicates the costs associated with the prevention of one additional recurrence for immediate versus delayed MMC instillation. The bootstrapped cost-effect pairs were plotted on cost-effectiveness planes to graphically show the uncertainty around the ICERs [19]. Cost-effectiveness acceptability curves were estimated to show the probability that immediate MMC instillation is cost-effective compared to delayed MMC instillation for a range of willingness-to-pay (WTP) thresholds [19]. WTP is defined as the amount of money that society is willing to invest for one additional month without a recurrence or for one additional recurrence prevented.

#### 2.4.3 Sensitivity analyses

First, we performed a complete case analysis in which cases with missing data were excluded. Second, we performed an unadjusted analysis in which no confounders were included. Third, we performed an analysis in which costs of delayed MMC instillation were excluded. This is a better representation of clinical practice, as delayed MMC instillation was introduced in the trial to ensure that all patients in a specific risk group received the same number of instillations and that only the timing of postoperative MMC instillation differed between the treatment arms. In clinical practice, however, delayed postoperative MMC instillation is not administered.

Stata v13 (StataCorp, College Station, TX, USA) was used for statistical analyses and for construction of graphs. Tests were two-sided and *p* < 0.05 was considered statistically significant.

## 3 Results

All 2243 NMIBC patients were analyzed in this economic evaluation. Baseline characteristics were comparable between the treatment arms (Table 2). The median age was 68 yr (IQR 60–74) and most patients were male (82%). Patients who did not experience an event of interest were followed during a median period of 32 mo (IQR 17–51).

**Table 2:** Baseline characteristics for the overall cohort and for the immediate and delayed MMC instillation groups

Characteristic	All patients (*n* = 2243)	iMMC (*n* = 1048)	dMMC (*n* = 1195)	*p*-value
Median age, yr (interquartile range)	68 (60–74)	68 (60–74)	68 (60–75)	NS
Sex, *n* (%)				NS
Male	1838 (82)	844 (81)	994 (83)	
Female	404 (18)	203 (19)	201 (17)	
Missing	1 (0)	1 (0)	0 (0)	
Primary/recurrence, *n* (%)				NS
Primary	1442 (64)	674 (64)	768 (64)	
Recurrence	801 (36)	374 (36)	427 (36)	
Number of tumors, *n* (%)				NS
Single	972 (43)	467 (45)	505 (42)	
Multiple	1271 (57)	581 (55)	690 (58)	
Tumor stage, *n* (%)				NS
Ta	1669 (74)	794 (76)	875 (73)	
T1	554 (25)	245 (23)	309 (26)	
Missing	20 (1)	9 (1)	11 (1)	
Tumor grade, *n (%)*				NS
Grade 1	856 (38)	406 (39)	450 (38)	
Grade 2	1031 (46)	491 (47)	540 (45)	
Grade 3	338 (15)	144 (14)	194 (16)	
Missing	18 (1)	7 (1)	11 (1)	
Carcinoma in situ, *n* (%)				NS
No	2181 (97)	1021 (97)	1160 (97)	
Yes	62 (3)	27 (3)	35 (3)	

NS = not significant; MMC = mitomycin C.

### 3.1 Effects and costs

There were 368/1048 recurrences (35%) after immediate MMC instillation and 521/1195 (44%) after delayed MMC instillation at 3-yr follow-up. Progression occurred in 33/1048 patients (3%) in the immediate MMC arm and 72/1195 patients (6%) in the delayed MMC arm at 3-yr follow-up. Time to recurrence was significantly longer for immediate MMC (27.31 mo) than for delayed MMC instillation (24.97 mo), with an adjusted mean difference of 2.21 mo (95% CI 1.58–2.84; Table 3). The proportion of patients with recurrence-free survival was significantly higher after immediate MMC instillation than after delayed MMC instillation (0.65 vs 0.56; adjusted mean difference 0.08, 95% CI 0.06–0.11).

**Table 3:** Mean effects and costs per patient for the iMMC and dMMC instillation arms and mean differences in effects and costs between the arms

Effects and costs	Mean (standard error)		Unadjusted difference	Adjusted difference a
	iMMC (*n* = 1048)	dMMC (*n* = 1195)	(95% CI)	(95% CI)
**Effects**				
Time to recurrence (mo) b	27.31 (0.43)	24.97 (0.42)	2.33 (1.71–2.95)	2.21 (1.58–2.84)
Recurrence-free survival (proportion)	0.65 (0.02)	0.56 (0.02)	0.09 (0.06–0.11)	0.08 (0.06–0.11)
**Costs**^b^				
TURBT (€)	4994 (62)	5287 (66)	−293 (−384 to −202)	−277 (−367 to −188)
Hospitalization after TURBT (€)	655 (8)	694 (9)	−38 (−50 to −27)	−36 (−48 to −25)
Immediate MMC instillation (€)	838 (0)	0 (0)	838	838
Delayed MMC instillation (€)	0 (0)	838 (0)	−838	−838
Cystoscopy (€)	5326 (46)	5292 (45)	35 (−59 to 128)	26 (−69 to 120)
Adjuvant instillations (€)	10 173 (231)	10 769 (189)	−596 (−903 to −289)	−370 (−676 to −64)
Electrocauterization (€)	27 (5)	31 (5)	−4 (−4 to −4)	−5 (−5 to −5)
Cystectomy + bLND (€)	599 (149)	1139 (190)	−539 (−887 to −192)	−486 (−840 to −132)
Hospitalization after cystectomy + bLND (€)	169 (42)	322 (54)	−152 (−250 to −54)	−138 (−237 to −37)
Computed tomography scan (€)	176 (24)	252 (28)	−77 (−126 to −27)	−64 (−114 to −13)
Total (€)	22 959 (339)	24 624 (352)	−1665 (−2117 to −1212)	−1350 (−1799 to −900)

CI = confidence interval; MMC = mitomycin C; dMMC = delayed MMC; iMMC = immediate MMC; TURBT = transurethral resection of bladder tumor; bLND = bilateral lymph node dissection.

a Analyses were adjusted for age, concomitant carcinoma in situ, gender, primary/recurrence, tumor grade, tumor multiplicity and tumor stage.

b In the second and third follow-up years, costs were discounted at an annual rate of 4% and effects were discounted at an annual rate of 1.5%.

Over a 3-yr time period, total mean health care costs per patient were significantly lower for immediate MMC than for delayed MMC (€22 959 vs €24 624; adjusted mean difference −€1350, 95% CI −€1799 to −€900; Table 3). All costs, except for cystoscopies, were significantly lower in the immediate MMC arm. Adjuvant instillations (adjusted mean difference −€370, 95% CI −€676 to −€64) and cystectomies (adjusted mean difference −€486, 95% CI −€840 to −€132) contributed most to the total cost difference.

### 3.2 Cost-effectiveness analyses

**Fig. 2:**
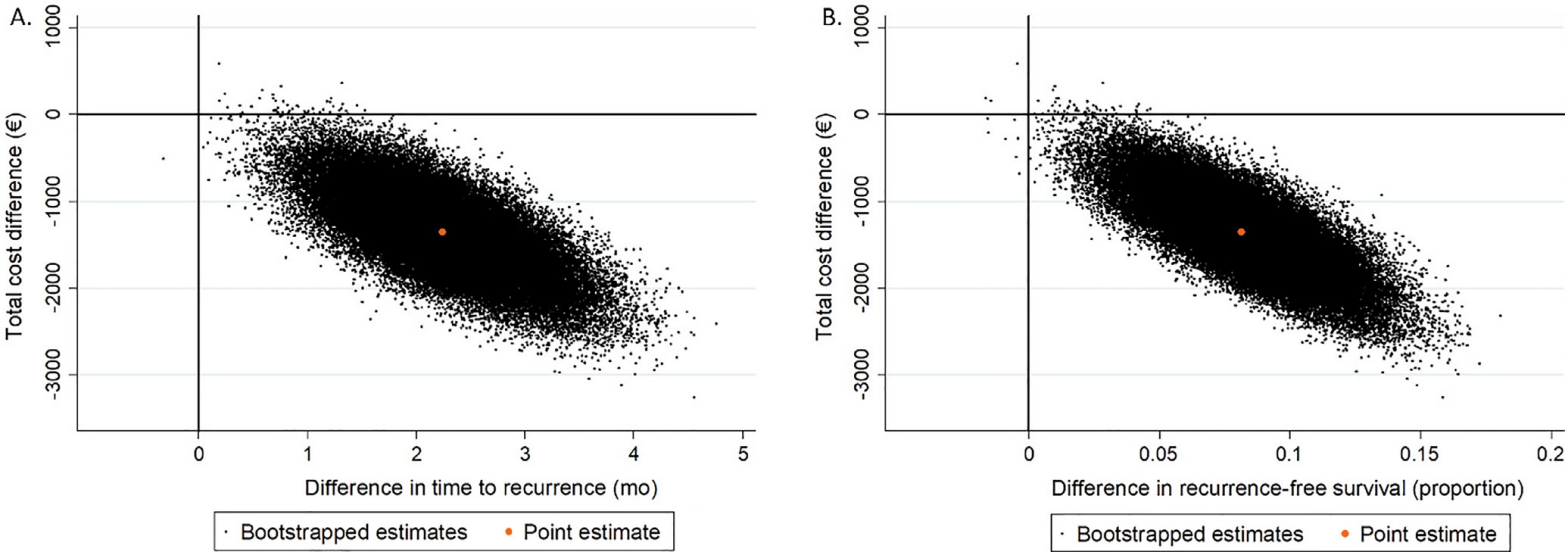
Cost-effectiveness planes showing the uncertainty around the incremental cost-effectiveness ratios for immediate mitomycin C (MMC) instillation compared to delayed MMC instillation for (A) time to recurrence and (B) recurrence-free survival.

For time to recurrence, the ICER was −611, indicating that one additional month without recurrence was associated with cost savings of €611 for immediate compared to delayed MMC instillation. For recurrence-free survival, the ICER was −16 547, indicating that one additional recurrence prevented resulted in cost savings of €16 547 for immediate versus delayed MMC instillation. Thus, immediate MMC was dominant over delayed MMC for both for time to recurrence and recurrence-free survival. Accordingly, the large majority of the bootstrapped cost-effect pairs were situated in the southeast quadrant of the cost-effectiveness plane for both outcomes (Fig. 2A,B). The probability of immediate MMC instillation being cost-effective compared to delayed MMC instillation for time to recurrence and for recurrence-free survival was 0.95 at all possible ceiling ratios.

### 3.3 Sensitivity analyses

In the complete case analysis, immediate MMC instillation was dominant over delayed MMC instillation for time to recurrence and for recurrence-free survival (Table 4). Results for the unadjusted analysis were similar to those for the main analysis. The unadjusted analysis showed larger differences in effects and costs between immediate and delayed MMC instillation. In both sensitivity analyses, the probability of cost-effectiveness was ≥0.95 at all possible ceiling ratios.

**Table 4:** Effect and cost differences between the iMMC and dMMC arms in the main and sensitivity analyses and corresponding ICERs and WTP for pCE of 0.95 and 0.88 for iMMC versus dMMC

Statistical analysis	iMMC	dMMC	Effect difference	Cost difference,	ICER	WTP (€ per AEU)	
	(*n*)	(*n*)	(95% CI)	€ (95% CI)	(€ per AEU)	pCE 0.95	pCE 0.88
**Main analysis**^a^							
Time to recurrence (mo)	1048	1195	2.21 (1.58–2.84)	−1350 (−1799 to −900)	−611 (dominant)	0	Dominant
Recurrence-free survival (proportion)	1048	1195	0.08 (0.06–0.11)	−1350 (−1799 to −900)	−16 547 (dominant)	0	Dominant
**Sensitivity analyses**							
Complete case analysis a							
Time to recurrence (mo)	636	752	2.58 (1.34–3.83)	−1339 (−2244 to −434)	−519 (dominant)	0	Dominant
Recurrence-free survival (proportion)	636	752	0.10 (0.05–0.15)	−1339 (−2244 to −434)	−13 386 (dominant)	0	Dominant
Unadjusted analysis							
Time to recurrence (mo)	1048	1195	2.33 (1.71–2.95)	−1665 (−2117 to −1213)	−714 (dominant)	0	Dominant
Recurrence-free survival (proportion)	1048	1195	0.09 (0.06–0.11)	−1665 (−2117 to −1213)	−19 558 (dominant)	0	Dominant
Analysis excluding dMMC instillation costs a							
Time to recurrence (mo)	1048	1195	2.21 (1.58–2.84)	−512 (−961 to −62)	−231 (dominant)	140	0
Recurrence-free survival (proportion)	1048	1195	0.08 (0.06–0.11)	−512 (−961 to −62)	−6274 (dominant)	4200	0

CI = confidence interval; ICER = incremental cost-effectiveness ratio; MMC = Mitomycin C; iMMC = immediate MMC; dMMC = delayed MMC; AEU = additional effect unit; WTP = willingness to pay; pCE = probability of being cost-effective; dominant = the probability of iMMC being cost-effective is at least 0.95 at any WTP threshold.

a Analyses were adjusted for age, concomitant carcinoma in situ, gender, primary/recurrence, tumor grade, tumor multiplicity, and tumor stage.

After costs of delayed MMC instillation were excluded, the probability of immediate MMC instillation being cost-effective was 0.88 at a WTP of €0 per additional month without recurrence and per additional recurrence prevented. The probability of cost-effectiveness increased to 0.95 at a WTP of €140 per additional month without recurrence and at a WTP of €4200 per additional recurrence prevented (Supplementary Fig. 1A,B).

## 4 Discussion

This trial-based economic evaluation showed that immediate MMC instillation was more effective and less expensive than delayed MMC instillation. This indicates that immediate MMC instillation is cost-effective. Our results are in line with previous model-based economic evaluations, which all concluded that immediate MMC instillation would result in cost savings [7], [8], [9], [10]. In addition, our analysis demonstrated that immediate MMC instillation is cost-effective regardless of the adjuvant treatment a patient receives afterwards.

In the present study, we found that costs related to progression contributed most to the cost difference between the immediate and delayed MMC instillation arms. However, owing to the longer time to recurrence in the immediate MMC arm, the risk of progression may have been underestimated in this treatment arm during the 3-yr follow-up period. Consequently, the cost-effectiveness of immediate MMC instillation may have been overestimated. Hence, the literature offers no evidence that immediate MMC instillation has a beneficial effect on progression [20]. Costs for cystoscopies did not contribute to the cost difference between the treatment arms. During the trial protocol, follow-up cystoscopies were similar in both treatment arms and for all NMIBC risk groups. Consequently, any differences in follow-up cystoscopies between the treatment arms could only arise after patients experienced a recurrence. The difference in time to recurrence between the treatment arms (27.31 vs 24.97 mo for immediate vs delayed MMC, adjusted mean difference 2.21 mo, 95% CI 1.58–2.84) presumably did not result in significantly lower costs for cystoscopies in the immediate MMC instillation arm during the 3-yr follow-up period.

Despite the evidence showing the effectiveness of immediate MMC instillation for patients with NMIBC, a recently published review revealed that adherence to administration of immediate MMC instillation is low [4], [21]. Adherence to immediate MMC instillation was only 43% in Europe and Australia, and even lower at 0.5% in the USA and Canada [21]. Our findings suggest that better adherence to immediate MMC instillation would result in substantial health gains and cost savings.

This study has several strengths. Effects and costs were estimated using individual patient data from an RCT with a large sample size. In addition, the validity of our results was explored in several sensitivity analyses. In one of these sensitivity analyses, we excluded costs of delayed MMC instillation, as this better represents clinical practice. This analysis showed that the probability that immediate MMC instillation is cost-effective compared to delayed MMC installation is 0.88 at a WTP of €0 per additional month without recurrence and per additional recurrence prevented. However, we could not exclude the possibility that delayed MMC instillation has no clinical benefit, meaning that effect differences may have been larger if delayed MMC instillation truly was not administered. Therefore, our results may be conservative. This study was limited by the rate of missing effect data and the retrospective estimation of costs based on expected treatment and follow-up schedules. Another limitation is that we mainly used mean prices for diagnosis-treatment combinations and the uncertainty around cost differences may therefore be underestimated [12]. Moreover, patients were stratified into risk groups that do not correspond to current EAU risk groups, and treatment and follow-up schedules differed from current EAU guidelines [4]. This may have had an impact on our findings, since the intensity of treatment and follow-up schedules, and therefore also costs, may have been underestimated or overestimated for some of the NMIBC patients. Since analyses were performed from a health care perspective, the costs of informal care or productivity losses were not considered, which may have led to underestimation of the cost differences. As some NMIBC patients are currently treated with adjuvant BCG instead of adjuvant MMC instillations, future research should evaluate the cost-effectiveness of immediate MMC instillation for these patients. Administration of adjuvant BCG instillations is expected to reduce overall costs for the treatment of NMIBC because of the lower risk of costly progression.

## 5 Conclusions

This trial-based cost-effectiveness analysis shows that from a health care perspective immediate MMC instillation is more effective and less expensive compared to delayed MMC instillation.

  ***Author contributions***: Jakko A. Nieuwenhuijzen had full access to all the data in the study and takes responsibility for the integrity of the data and the accuracy of the data analysis.

*Study concept and design*: Hentschel, van Moorselaar, Bosmans, Nieuwenhuijzen.

*Acquisition of data*: Bosschieter, Hentschel, Blankvoort.

*Analysis and interpretation of data*: Bosschieter, Hentschel, Blankvoort, Bosmans, Nieuwenhuijzen.

*Drafting of the manuscript*: Hentschel, Blankvoort, Bosmans, Nieuwenhuijzen.

*Critical revision of the manuscript for important intellectual content*: Hentschel, Blankvoort, Bosschieter, Vis, van Moorselaar, Bosmans, Nieuwenhuijzen.

*Statistical analysis*: Bosschieter, Hentschel, Blankvoort, Bosmans.

*Obtaining funding*: None.

*Administrative, technical, or material support*: Hentschel, Blankvoort.

*Supervision*: Vis, van Moorselaar, Bosmans, Nieuwenhuijzen.

*Other*: None.

  ***Financial disclosures:*** Jakko A. Nieuwenhuijzen certifies that all conflicts of interest, including specific financial interests and relationships and affiliations relevant to the subject matter or materials discussed in the manuscript (eg, employment/affiliation, grants or funding, consultancies, honoraria, stock ownership or options, expert testimony, royalties, or patents filed, received, or pending), are the following: None.

  ***Funding/Support and*** role ***of the sponsor*:** None.
