# Supplementary material for: Trial-based Cost-effectiveness Analysis of an Immediate Postoperative Mitomycin C Instillation in Patients with Non–muscle-invasive Bladder Cancer
Source: Eur Urol Open Sci. 2022 Jan 17;37:7–13. doi: 10.1016/j.euros.2021.12.008 (PMC8883187; doi:10.1016/j.euros.2021.12.008)
Supplement: Supplementary data 1 [file mmc1.docx]

**Supplementary Table 1.** Since follow-up was by design terminated after first recurrence of disease, we needed to make assumptions on the follow-up after first recurrence of disease. Disease could recur as intermediate- or high-risk non-muscle-invasive bladder cancer (NMIBC) or could progress to muscle-invasive bladder cancer (MIBC). This table provides an overview of the assumptions that were made on the treatment and follow-up schedules of intermediate- and high-risk NMIBC patients and of MIBC patients. The tumor characteristics, treatment and follow-up schedules are in accordance with the European Association of Urology guidelines [1].

|  | **Intermediate-risk NMIBC** | **High-risk NMIBC** | **MIBC** |
| --- | --- | --- | --- |
| **Tumor characteristics** | Recurrent NMIBC with no characteristics of high-risk NMIBC | T1; G3 (HG); CIS; multiple, recurrent and >3cm TaG1-G2 (LG) | ≥T2 |
| **Treatment schedule** | Mitomycin C instillations for 6 months | Bacillus Calmette-Guérin instillations for three years | Radical cystectomy and bilateral lymph node dissection |
| **Follow-up schedule** | Cystoscopy every 3 months (year 1)  Cystoscopy every 4 months (year 2)  Cystoscopy every 6 months (year 3) | Cystoscopy every 3 months (year 1 – 2)  Cystoscopy every 6 months (year 3)  CT scan every 12 months (year 1 – 3) | CT scan every 4 months (year 1)  CT scan every 6 months (year 2 – 3) |
| Abbreviations: CIS: carcinoma in situ; CT: computed tomography; G1-3: grades 1-3; MIBC: muscle-invasive bladder cancer; NMIBC: non-muscle-invasive bladder cancer;. | | | |

**Supplementary Figure 1.** Cost-effectiveness acceptability curves for (A) time to recurrence and for (B) recurrence-free survival visualizing the probability of immediate post-operative Mitomycin C (MMC) instillation being cost-effective compared with delayed MMC instillation for a range of willingness-to-pay t
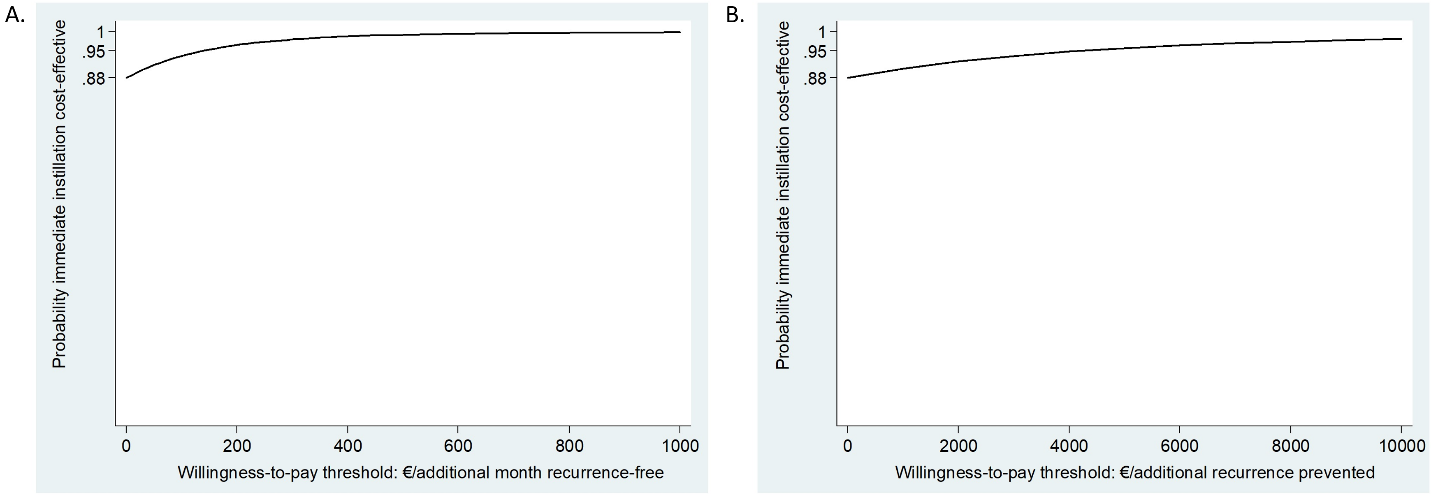
hresholds. Costs of delayed MMC instillation were excluded.

**REFERENCES**

1. Babjuk M, Burger M, Comperat EM, et al. European Association of Urology Guidelines on Non-muscle-invasive Bladder Cancer (TaT1 and Carcinoma In Situ) - 2019 Update. Eur Urol. 2019;76(5):639-57.
